# A Nomogram for Pretreatment Prediction of Response to Induction Chemotherapy in Locally Advanced Hypopharyngeal Carcinoma

**DOI:** 10.3389/fonc.2020.522181

**Published:** 2020-12-11

**Authors:** Baoliang Guo, Fusheng Ouyang, Lizhu Ouyang, Xiyi Huang, Haixiong Chen, Tiandi Guo, Shao-min Yang, Wei Meng, Ziwei Liu, Cuiru Zhou, Qiu-gen Hu

**Affiliations:** ^1^Department of Radiology, Shunde Hospital of Southern Medical University, Foshan, China; ^2^Department of Clinical Laboratory, The Affiliated Shunde Hospital of Guangzhou Medical University, Foshan, China

**Keywords:** advanced hypopharyngeal carcinoma, induction chemotherapy, nomogram, prediction, response

## Abstract

**Background:**

Induction chemotherapy (IC) significantly improves the rate of larynx preservation; however, some patients could not benefit from it. Hence, it is of clinical importance to predict the response to IC to determine the necessity of IC. We aimed to develop a clinical nomogram for predicting the treatment response to IC in locally advanced hypopharyngeal carcinoma.

**Methods:**

We retrospectively include a total of 127 patients with locally advanced hypopharyngeal carcinoma who underwent MRI scans prior to IC between January 2014 and December 2017. The clinical characteristics were collected, which included age, sex, tumor location, invading sites, histological grades, T-stage, N-stage, overall stage, size of the largest lymph node, neutrophil-to-lymphocyte ratio, hemoglobin concentration, and platelet count. Univariate and multivariate logistic regression was used to select the significant predictors of IC response. A nomogram was built based on the results of stepwise logistic regression analysis. The predictive performance and clinical usefulness of the nomogram were determined based on the area under the curve (AUC), calibration curve, and decision curve.

**Results:**

Age, T-stage, hemoglobin, and platelet were four independent predictors of IC treatment response, which were incorporated into the nomogram. The AUC of the nomogram was 0.860 (95% confidence interval [CI]: 0.780-0.940), which was validated using 3-fold cross-validation (AUC, 0.864; 95% CI: 0.755-0.973). The calibration curve demonstrated good consistency between the prediction by the nomogram and actual observation. Decision curve analysis shows that the nomogram was clinically useful.

**Conclusion:**

The proposed nomogram resulted in an accurate prediction of the efficacy of IC for patients with locally advanced hypopharyngeal carcinoma.

## Introduction

Hypopharyngeal carcinoma is an uncommon tumor, which accounts for approximately 3%–5% of mucosal head and neck subsites (1). More than 80% of hypopharyngeal tumors arise from the pyriform sinus, which is the most common subsite, and 20% arise from the posterior pharyngeal wall and postcricoid region (2). Over 2/3 of patients present with locally advanced stages of the disease (3). Although some progress has been achieved in treatment management, patients with advanced hypopharyngeal carcinoma still have a poor prognosis with a 5-year overall survival rate of only 25%–40% (4). Laryngectomy and pharyngeal reconstruction has historically been the primary treatment for this disease (5). In recent years, laryngeal-preservation approaches have been developed to preserve speech and swallowing function. Clinically, induction chemotherapy (IC) with docetaxel, cisplatin, 5-fluorouracil (TPF) is regarded as a strong predictor of radiosensitivity and the landmark treatment of nonsurgical larynx-preservation approaches (6). TPF-based IC has been proved to significantly improve the rate of larynx preservation. Identifying good responders to IC is considered the preferred modality of larynx preservation.

Pretreatment prediction of response to IC in patients with advanced hypopharyngeal carcinoma is crucial for patient stratification and further precise treatment. Resistance to chemoradiotherapy is widely recognized as the main cause of recurrence in locally advanced hypopharyngeal cancer. Recently, researchers have been seeking new predictors of chemoradiotherapy sensitivity to determine possibilities for larynx preservation. Although IC before concurrent radiochemotherapy has not been routinely applied in the clinical setting, it may serve as a prognostic tool with the potential to change subsequent therapy depending on the response (7). Oh et al. find that radiomic features derived from 18-fluorodeoxyglucose (^18^F-FDG) positron emission tomography (PET) images of hypopharyngeal carcinoma patients could evaluate their response to IC (8). Emerging evidence suggests that diffusion-weighted imaging (DWI) MRI improves tissue characterization, staging, and response to therapy in those with head and neck carcinoma (9–14). Noij et al. compare the efficacy of FDG-PET/CT and DWI MRI in evaluating the IC response of the primary tumor and find that the FDG-PET/CT had better performance with a specificity of 86.5% and sensitivity of 85.7% (15). Guo et al. indicate that pretreatment Intra-Voxel Incoherent Motion (IVIM) DWI can potentially predict the treatment response to IC in those with advanced hypopharyngeal carcinoma (16). Studies on various molecular markers have been undertaken, such as Ki-67 antigen, p53 protein, and epidermal growth factor receptor (EGFR) (17). Some of studies show that only EGFR is a favorable predictor for response to IC, whereas others do not (17). To date, the selection criteria for patients who may benefit from IC treatment remains inadequate. Nonetheless, it is meaningful to predict the response to IC to optimize individual treatment regimens.

We hypothesize that a quantitative tool incorporating useful clinical data could help predict the response to IC in patients with hypopharyngeal cancer. Therefore, we aimed to develop a nomogram based on patient and tumor characteristics to select patients who are sensitive to IC and potential candidates for laryngeal preservation.

## Materials and Methods

### Patients

This retrospective study was approved by the ethics committee of the first author’s institution, and the requirement for patient informed consent was waived. A total of 127 patients with pathologically confirmed hypopharyngeal squamous cell carcinoma were identified from the records of the Institutional Picture Archiving and Communication System (PACS, Carestream) between January 2014 and December 2017 in our department. All patients had undergone MRI scans and laboratory tests prior to IC treatment. Patients were administered a treatment regimen that comprised cisplatin (P) and 5-fluorouracil (F) with or without docetaxel (T), and they had undergone routine follow-up during three cycles of IC. We excluded patients who discontinued the IC due to adverse reactions.

### Clinical Characteristics

The pretreatment clinical characteristics were collected, which included patient age, sex, tumor location (pyriform sinus, posterior hypopharyngeal wall, or postcricoid region), invasion sites (esophagus, tongue root, thyroid cartilage, multiple, or none), histological grades (low, intermediate, or high grade), T stage (T1, T2, T3, or T4), N stage (N0, N1, N2, or N3), overall stage (I–IV), size of the largest cervical lymph node (measured in the short-axis plane), neutrophil-to-lymphocyte ratio (NLR), hemoglobin (HGB) (g/L), and platelet (PLT) (× 10^9^/L). Histological grade was determined by the biopsy specimen. We classified well-differentiated grade as low grade, moderately differentiated grade as intermediate grade, and poorly differentiated grade as high grade. Tumor staging was reassessed according to the AJCC Staging System Manual, 8th Edition. The patient and tumor characteristics were reviewed by two independent radiologists with more than 10 years of experience in head and neck cancer. Disagreements between the two radiologists were resolved by consensus and, if necessary, a consultation with a third radiologist.

### Follow-Up and Treatment Response Assessment

All patients underwent a follow-up assessment after receiving three cycles of IC. The Response Evaluation Criteria in Solid Tumors was used to evaluate the tumor response to IC regimen. The treatment response to IC was assessed by follow-up MRI. We defined complete response (CR) and partial response (PR) as responsive, whereas stable disease (SD) and progression disease (PD) were categorized as nonresponsive. The primary endpoint of this study was the treatment response to IC.

### MRI Imaging Acquisition

MRI scans were performed on a 3.0T SIEMENS Skyra system (Siemens Medical Solutions). The scanning parameters were as follows for the T1-weighted turbo-spin-echo (TSE) sequence: TR/TE, 690/9.4 ms; FOV, 240 mm × 240 mm; slice thickness, 3 mm; slice gap, 0.3 mm. The scanning parameters were as follows for the T2-weighted TSE sequence: TR/TE, 5050/78 ms; FOV, 240 mm × 240 mm; slice thickness, 3 mm; slice gap, 0.3 mm. The axial fat-suppressed T2-weighted TSE sequence parameters were as follows: TR/TE, 4550/63 ms; FOV, 240 mm × 240 mm; slice thickness, 3 mm; slice gap, 0.3 mm. The imaging parameters for the gadolinium-DTPA-enhanced T1-weighted TSE sequences with fat saturation were as follows: TR/TE, 710/11 ms; FOV, 240 mm × 240 mm; slice thickness, 3 mm; slice gap, 0.3 mm.

### Statistical Analysis

All statistical calculations were computed using R software (version 2.3.2). To identify predictors for IC response, variables with *p*-value <0.10 in univariable logistic regression analysis were entered into multivariate logistic regression analysis. A nomogram was built based on the results of the stepwise logistic regression analysis. The nomogram is based on proportionally transforming the regression coefficient into a 0- to 100-point scale. The sum of the points from all variables could be interpreted into a probability of belonging to a class. The predictive performance of the nomogram was measured by the AUC, sensitivity, and specificity in a 3-fold cross-validation setting. The model calibration was assessed by calibration curve and Hosmer-Lemeshow test. Decision curve analysis (DCA) was performed to evaluate the clinical usefulness of the nomogram by calculating the net benefits at different threshold probabilities (0%–100%) in the combined primary and validation data sets. The formulation and calibration of the nomogram were applied using the “rms” package. DCA was done using the “rmda” package. A two-tailed *P* <0.05 was considered to be statistically significant.

## Results

### Patient and Tumor Characteristics

The mean age of the 127 patients was 58.1 years ± 9.6, and 121 (95.3%) were males. A total of 52 (40.9%) patients received the PF regimen, and 75 (59.1%) received the TPF regimen. The number of responses to IC in patients who received PF and TPF was 31 (59.6%) and 40 (53.3%) with no significant difference (*p* = 0.483). **Table 1** shows the comparison of patient and tumor characteristics between the responsive and nonresponsive groups.

**Table 1:** Patient and tumor characteristics in primary and validation cohorts.

	Response(n = 71)	Nonresponse(n = 56)	P-value
**Sex, No. (%)**			
Male	67 (94.4)	54 (96.4)	0.694
Female	4 (5.6)	2 (3.6)	
**Age, mean ± SD, years**	60.3 ± 9.6	55.5 ± 9.0	0.005
**Tumor Location, No. (%)**			
Pyriform sinus	56 (78.9)	44 (78.6)	0.282
Posterior pharyngeal wall	5 (7.0)	8 (14.3)	
Postcricoid region	9 (12.7)	4 (7.1)	
**Invasion sites, No. (%)**			
Esophagus	12 (16.9)	7 (12.5)	0.089
Tongue root	0	3 (5.4)	
Thyroid cartilage	18 (25.4)	20 (35.7)	
Multiple	37 (52.1)	25 (44.6)	
None	4 (5.6)	1 (1.8)	
**Histological grade, No. (%)**			
Low grade	6 (8.5)	11 (19.6)	0.159
Intermediate grade	46 (64.8)	34 (60.7)	
High grade	19 (26.8)	11 (19.6)	
**T stage, No. (%)**			
1	5 (7.0)	1 (1.8)	0.071
2	25 (35.2)	12 (21.4)	
3	16 (22.5)	12 (21.4)	
4	25 (35.2)	31 (55.4)	
**N stage, No. (%)**			
0	8 (11.3)	1 (1.8)	0.123
1	15 (21.1)	17 (30.4)	
2	46 (64.8)	36 (64.3)	
3	2 (2.8)	2 (3.6)	
**Overall stage, No. (%)**			
3	19 (26.8)	20 (35.7)	0.594
4	52 (73.2)	36 (64.3)	
**LN size (cm), mean ± SD**	1.93 ± 1.39	1.81 ± 0.87	0.566
**NLR (%), mean ± SD**	3.16 ± 1.93	3.27 ± 2.15	0.786
**HGB (g/L)**	140.4 ± 12.8	125.4 ± 13.1	<0.001
**PLT (× 10^9^/L)**	244.3 ± 59.1	279.0 ± 93.5	0.012

T, tumor; N, node; LN, Lymph node; NLR, neutrophil lymphocyte ratio; HGB, hemoglobin; PLT, platelet; SD, standard deviation.

### Prediction Model Development and Validation

**Figure 1:**
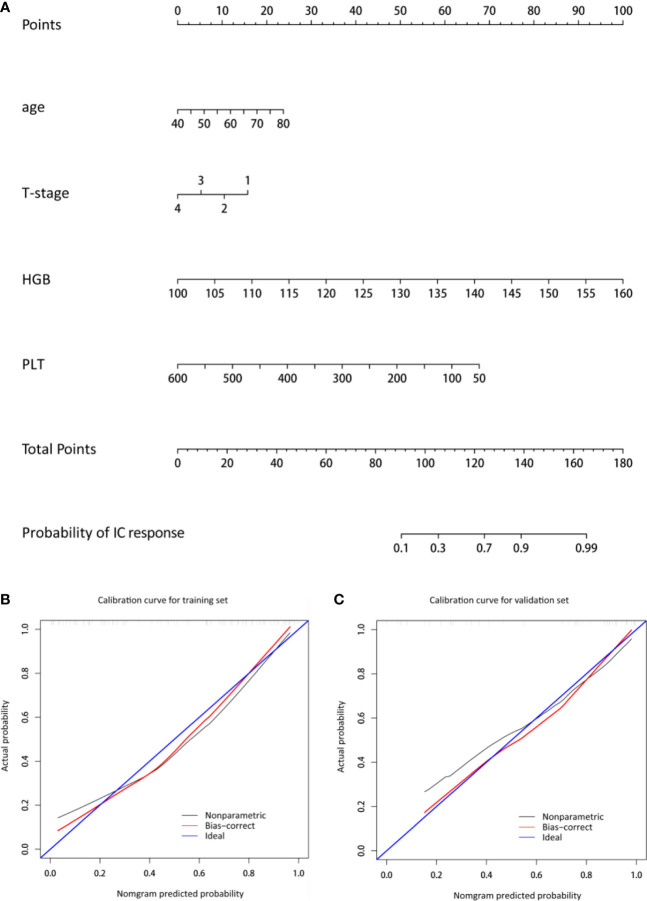
Nomogram and the corresponding calibration curve. **(A)** The nomogram was developed with age, T-stage, HGB, and PLT incorporated. **(B, C)** The calibration curves of the nomogram in the training and cross-validation data sets. The *x*- and *y*-axes are the nomogram-predicted probability and actual probability of IC response, respectively. The red line represents the performance of the nomogram, and the blue line represents a perfect prediction by an ideal model. A closer fit to the diagonal blue line represents a better prediction. HGB, hemoglobin; PLT, platelet.

Four predictors of IC response, including age, T stage, HGB, and PLT were selected (**Table 2**). **Figure 1A** shows the nomogram incorporating the abovementioned independent predictors. The nomogram for IC response prediction yielded an AUC of 0.860 (95% CI: 0.780-0.940), sensitivity of 72.3%, specificity of 86.5%, and an accuracy of 78.6% in the training data set. In the cross-validation data set, the nomogram achieved an AUC of 0.864 (95% CI: 0.755-0.973), sensitivity of 75.0%, specificity of 84.2%, and an accuracy of 79.1%. **Figures 1B, C** shows the calibration curve of the model, indicating good consistency between the prediction by the nomogram and actual observation (Hosmer-Lemeshow test, *p* = 0.257 in the training data set and 0.772 in the cross-validation data set).

**Table 2:** Risk factors associated with IC response in univariable and multivariable logistic regression analysis.

Characteristics	Univariable analysis		Multivariable analysis	
	OR (95% CI)	p-value	OR (95% CI)	p-value
Sex	4.077 (0.307-54.180)	0.287		
Age	1.079 (1.016-1.146)	0.014	1.055 (1.006-1.107)	0.029
Tumor location	1.797 (0.764-4.226)	0.179		
Histological grade	3.021 (1.191-7.662)	0.020		
T-stage	0.502 (0.279-0.904)	0.022	0.536 (0.330-0.871)	0.012
N-stage	0.233 (0.070-0.776)	0.018		
Overall stage	1.347 (0.411-4.419)	0.623		
LN size	2.585 (1.267-5.273)	0.009		
Invasion sites	0.983 (0.674-1.434)	0.930		
NLR	1.167 (0.878-1.552)	0.288		
HGB	1.144 (1.083-1.209)	<0.001	1.125 (1.072-1.179)	<0.001
PLT	0.988 (0.980-0.997)	0.007	0.991 (0.984-0.998)	0.017

OR, odds ratio; T, tumor; N, node; LN, Lymph node; NLR, neutrophil lymphocyte ratio; HGB, hemoglobin; PLT, platelet; SD, standard deviation.

### Clinical Usefulness of the Nomogram

**Figure 2:**
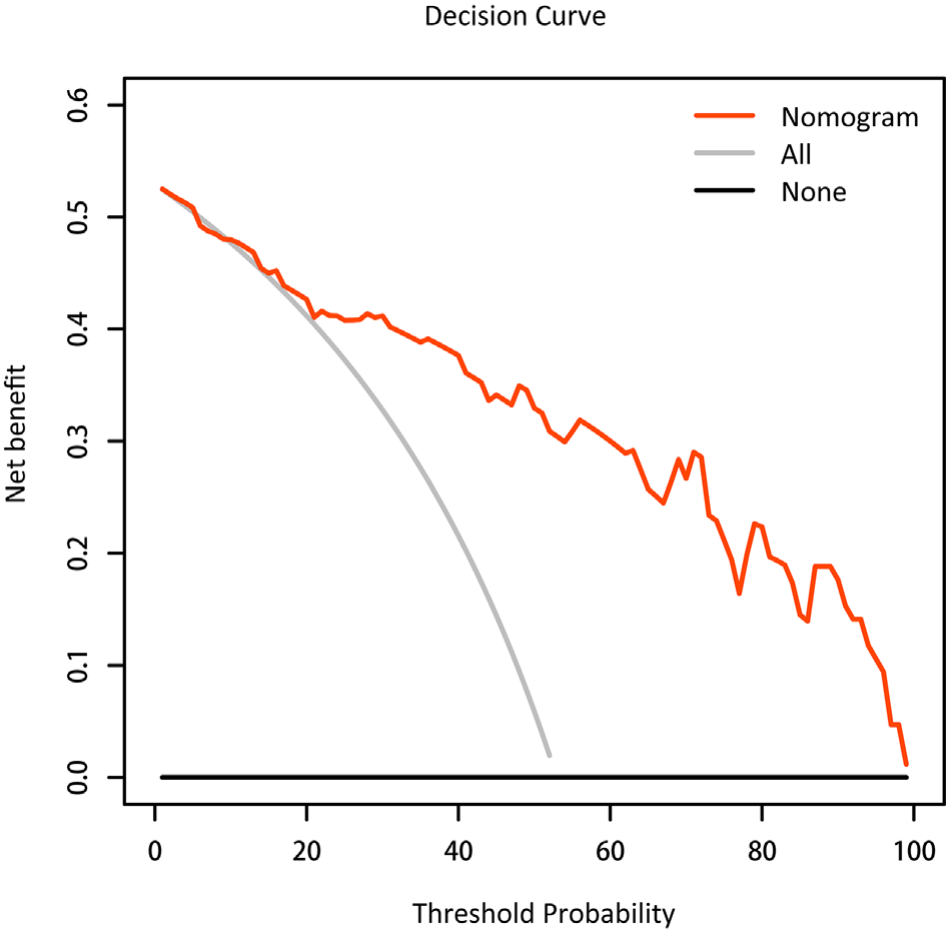
Decision curve analysis for the nomogram. The black and gray lines represent no and all patients that are responders, respectively. The net beneﬁt of IC treatment was calculated by subtracting the rate of patients with a false positive from the rate of those with a true positive, weighted by the relative harm of abandoning treatment compared with the negative results of an unnecessary treatment.

In the decision curve, the nomogram provided a net benefit of IC over the “treat-all” or “treat-none” strategy at a threshold probability >10% (**Figure 2**), indicating that the nomogram was useful. For example, the nomogram could offer an additional net benefit of 0.300, 0.270 as compared to the “treat-all” or “treat-none” strategy with a threshold probability of 60% and 70%, respectively.

## Discussion

We identified seven factors associated with IC response in patients with locally advanced hypopharyngeal cancer, which included patient age, T-stage, HGB, and PLT. The developed nomogram achieved good predictive performance with an AUC of 864 (95% CI: 0.755-0.973). The nomogram was proven to be clinically useful.

Head and neck carcinomas show a heterogeneous response to chemoradiotherapy with locoregional control and a 5-year OS rate ranging from below 50% to 80%. Few markers related to tumor response to therapy are already available for this tumor entity. Long-term survival evaluation and preservation of organ function are the two most important issues in the treatment of head and neck cancer. Although survival differs in subsites of head and neck cancer, it is usually poor in advanced stages (III or IV). Although numerous randomized trials suggested that IC may not improve patients’ survival rates, nonrandomized data indicate that IC may be helpful in preserving laryngeal function (such as phonatory speech). For patients with larynx and oropharyngeal cancers, the IC strategy could shrink or downstage tumors and, therefore, increase the laryngeal preservation rates and/or reduce the risk of locoregional recurrence and/or distant metastasis (18). Additionally, IC is able to predict a response to subsequent radiotherapy because significant correlations have been reported between the response to IC and success of subsequent radiotherapy (19). A large-scale phase III study shows that IC followed by definitive radiotherapy could preserve the larynx in 64% of patients in advanced laryngeal carcinoma (20). Comparable 2-year OS rate was observed in the organ preservation and surgical treatment arms (20).

IC has become an initial treatment for late-stage hypopharyngeal carcinoma; however, there are no data regarding IC sensitivity for this population. In clinical practice, treatment response assessment depends on conventional MRI to monitor tumor morphology changes. In contrast, functional imaging techniques, such as PET/CT, DWI MRI, and DCE MRI can reflect additional information about the underlying tumor biology, such as metabolic activity, cellularity, diffusion and perfusion. Based on the (18)F-FDG PET/CT images of hypopharyngeal cancers before treatment, a retrospective study obtained the first-order features, including standardized uptake value (SUV), metabolic tumor volume (MTV), and high-order textural features, such as coarseness, busyness, complexity, and contrast (8). As compared to nonresponders, responders had a lower maximum SUV, lower MTV, lower coarseness, and busyness (8). Compared with conventional imaging techniques, functional imaging techniques could provide an earlier evaluation of treatment response due to changes in tumor metabolism that often precede a reduction in tumor size. Timely assessment of treatment response may allow clinicians to shift patients away from ineffective to effective therapies as early as possible. However, optimal timing and interpretation criteria for the use of functional imaging techniques in daily practice have yet to be established (21). In addition, these imaging techniques could not predict the response to IC before treatment.

Studies on identifying predictors of treatment response to IC before treatment in patients with hypopharyngeal cancer are scarce. Sun et al. show that peripheral inflammation markers, including the pretreatment lymphocyte count, NLR, and platelet-to-lymphocyte ratio, are predictors of positive responses to IC in hypopharyngeal cancer patients (22). Suzuki et al. find that tumor location, nodal involvement, and pretreatment serum hemoglobin values are predictors for treatment response (23). Małecki et al. determine that the lack of EGFR expression instead of p53 and Ki-67 is a favorable predictor for IC response in patients with advanced hypopharyngeal cancer who are treated with a larynx-preservation treatment (17). In this present study, we identify the patients’ age, T-stage, HGB, and PLT as predictive indicators of treatment response to IC in locally advanced hypopharyngeal carcinoma. Older age, higher HGB, lower T-stage, and lower PLT are associated with a better response to IC. Luo et al. demonstrate that a response to IC is associated with lymph node size, tumor grade, invasion region, T-stage, and primary tumor site (24). Previous studies show that histological grade is correlated with chemotherapy response (25–27). Studies focused on other tumors select the HGB concentration as the single independent predictor of neoadjuvant chemoradiotherapy (28, 29). Huang et al. find that a PLT count ≥100 × 10^9^/L is an independent prognostic factor of complete remission (30). Based on previous studies, our nomogram based on the available clinical, imaging, and pathology data achieves high performance in predicting the treatment response to IC.

This study also has some limitations. First are the retrospective nature of this study and that it was performed in a single center. Second, there lacks external validation in a random patient set of advanced hypopharyngeal cancer. Third, about 63% of cases are pyriform cancers, which make the final outcomes more favorable to this subsite. Finally, the usability of the nomogram we constructed should be validated in a prospective cohort of patients.

In conclusion, this study screened four predictors of treatment response to an IC regimen in advanced hypopharyngeal carcinoma. A nomogram was built based on the factors to identify patients who are responders to IC treatment to avoid unnecessary therapy in nonresponding patients. DCA confirms the clinical usefulness of the nomogram.

## Data Availability Statement

All datasets generated for this study are included in the article/supplementary material.

## Ethics Statement

The studies involving human participants were reviewed and approved by Shunde Hospital of Southern Medical University. The ethics committee waived the requirement of written informed consent for participation.

## Author Contributions

Conception and design: BG, FO, QH. Acquisition of data: BG, FO, LO, XH, HC. Analysis and interpretation of data: BG, FO, TG, S-MY, WM, ZL, CZ. Drafting or revising the article: BG, FO, Q-gH. All authors contributed to the article and approved the submitted version.

## Funding

This research was supported by grants of the science and technology planning project of Foshan (2017AB003623, 2017AB003683); the Scientific Research Foundation for the Younger researchers of Shunde Hospital (SRSP2018010); the Scientific Research Foundation for the Younger researchers of Southern Medical University (PY2018N116); Guangdong Medical Science and Technology Research Fund (A2020395, A2020089), Foshan self-funded science and technology project (2017AB003623, 2017AB003683).

## Conflict of Interest

The authors declare that the research was conducted in the absence of any commercial or financial relationships that could be construed as a potential conflict of interest.
